# *topr*: an R package for viewing and annotating genetic association results

**DOI:** 10.1186/s12859-023-05301-4

**Published:** 2023-06-28

**Authors:** Thorhildur Juliusdottir

**Affiliations:** GeneDx Iceland, Katrinartun 4, 105 Reykjavík, Iceland

**Keywords:** GWAS, Association analysis, Visualisation, R package, Annotation, Manhattan

## Abstract

**Background:**

The successful identification of genetic loci for complex traits in genome-wide association studies (GWAS) has resulted in thousands of GWAS summary statistics becoming publicly available for hundreds of complex traits from multiple cohorts and studies. Visualisation is an important aid for interpreting, comparing, validating, and obtaining an overview of large amounts of data. However, the current software is limited in its ability and flexibility to annotate and simultaneously display multiple GWAS results which is useful when interpreting and comparing association results. Therefore, I created the *topr* R package to facilitate visualisation, annotation, and comparisons of single or multiple GWAS results. It contains functions tailored for viewing and analysing GWAS results.

**Results:**

*topr* provides a fast and elegant visual display of association results, along with the annotation of association peaks with their nearest gene. Association results from multiple analyses can be viewed simultaneously over the entire genome or in a more detailed regional view along with gene information. Users can perform the essential steps of visually exploring and annotating association results and generating elegant publication-ready plots.

**Conclusions:**

*topr* is developed as a package for the R statistical computing environment, released under the GNU General Public License, and is freely available on the Comprehensive R Archive Network (http://cran.r-project.org/package=topr). The source code is available at GitHub (https://github.com/totajuliusd/topr). *topr* provides several advantages and advances over the current alternatives, particularly in its gene annotation functionality and customisable display of single- or multiple-association results. With *topr*, I provide a flexible tool with multiple features to aid in the analysis and evaluation of GWAS association results.

## Background

Genome-wide association studies (GWAS) have successfully identified thousands of genetic loci for hundreds of complex traits [1]. The launch of large biobank projects across the world, including England, Finland, Iceland, Estonia, Japan, and the USA, and sharing of genetic data has been a major facilitator of GWAS, resulting in thousands of GWAS summary statistics becoming publicly available (the effect sizes and their standard errors or *p*-values on millions of SNPs) [2, 3]. Cross-biobank meta-analyses are becoming increasingly relevant for combining GWAS summary statistics from individual studies to boost sample size and, thereby, the power to detect associations [4].

When examining GWAS summary statistics, it is helpful to obtain a visual overview of the full results, followed by a more detailed regional view. It is especially useful when conducting cross-biobank studies and/or meta-analyses to obtain a clear overview of each contributing dataset and to visually evaluate their respective association peaks and compatibility.

Currently, there are several R packages available for plotting genetic association results; however, these packages do not provide inbuilt gene-annotation, the ability to directly compare and annotate multiple GWAS simultaneously and helper functions to simplify common operations in analysing association results. For example, the *qqman* package is widely used for generating basic Manhattan and Quantile–Quantile (QQ) plots [5]. *Manhattanly* is inspired by *qqman* and generates interactive versions of plots created by *qqman* [6]. The CMPlot package (now integrated into the GWAS package *rMVP*) can be used to generate Circular Manhattan plots as well as more traditional Manhattan plots displaying multiple GWAS results [7]. KaryoploteR offers visualisation of multiple GWAS results and more detailed regional view with gene information [8]. However, due to the greater complexity of KaryploteR, creating these plots requires multiple lines of code compared to a single line of code in *topr*. In addition, KaryoploteR is based on R’s base graphic system, whereas *topr* utilises ggplot2 and returns ggplot objects and can thus be used with other ggplot2 functions [9].

Therefore, I developed, and hereby present *topr,* a publicly available R package tailored for visualizing, annotating, and exploring GWAS results. A key advancement is the ability to easily compare and annotate multiple association results simultaneously. For example, with *topr* the user can display and visually compare multiple association results and then specify which peaks are annotated in each dataset according to various criteria, such as labelling density and association significance (e.g., label the top variant within each 1 Mb window with a *p*-value below 5 × 10^−8^). Additional advances of *topr* are that one can zoom into genetic regions using gene or variant names to view smaller regions of the genome, along with displaying gene information. Furthermore, helper functions are included to simplify common operations in analysing association results, such as the extraction of lead/top variants annotated with their nearest gene and identification of variants overlapping in two datasets with respect to genetic position and alleles.

### Implementation and general design

*topr* is written in the R programming language and utilises the *ggplot2* and *ggrepel* R graphics libraries for plotting. It is essentially the R-version of Toppar, a customisable database-driven browser for GWAS results [10]. *topr* comes with three example GWAS summary statistics datasets, one on Ulcerative Colitis retrieved from the UK biobank, and the other two on Crohn’s disease obtained from the FinnGen and UK biobanks, respectively (see the website for more details). *G*ene and exon datasets are imported on installation from the *toprdata* package, which includes genome builds GRCh38 and GRCh37 from Ensembl. *topr* has been tested on macOS, Linux, and Windows operating systems. It is freely available on the Comprehensive R Archive Network (http://cran.r-project.org/package=topr), and can be installed from within any R console or editor using the *install.packages* function. *topr´s* two key plot functions, *manhattan* and *regionplot,* display GWAS results over the entire genome and for smaller genetic regions along with gene information. Also included in *topr* as *locuszoom*, is a LocusZoom [11] like plot, commonly used in GWA studies to display regional linkage disequilibrium (LD) patterns between selected variants. The three plot functions require as their first argument either a single association dataset or multiple association datasets (represented as a single list in R), where each dataset must include at least three columns (CHROM, POS, and P), and the exact order and naming of the columns is flexible (i.e., the chr label can be either chr or chrom and is case-insensitive). The *locuszoom* plot function requires an additional column with a precalculated variant correlation (r^2^) value in the input datasets.

**Fig. 1:**
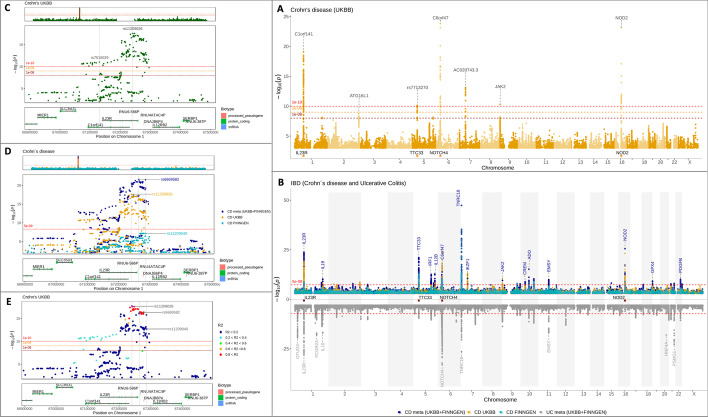
Regional and Manhattan plots of single and multiple GWAS results generated using *topr*´s plotting functions. A single GWAS *manhattan* plot **A** showing Crohn’s disease association over the entire genome, with index/lead variants (top variant per 1 Mb window), with *p*-values below 5 × 10^−9^ annotated with their nearest genes. A multi-GWAS *manhattan* plot **B** displaying three association results for Crohn´s disease on the top panel and association results for Ulcerative Colitis on the bottom panel. The top plot shows Crohn´s disease associations from UKBB (orange) and FinnGen (turquoise), along with their combined (meta) association results (dark blue). An example *regionplot* **C** zoomed in on the IL23R gene, showing associations to Crohn´s disease in one of *topr*´s example datasets. The two variants are labelled with their rsid´s and vertical lines highlight their positions in relation to the genes below. The horizontal lines in the main plot represent three different significance thresholds. By default, a significance threshold of − log10(5 × 10^−8^) is displayed on all plots. The line can be removed, altered or multiple thresholds shown. A multi GWAS regional plot **D** displaying Crohn’s disease association over the IL23R gene in three different datasets (UKBB, Finngen and combined meta) with the top variant within the region for each dataset highlighted by their variant id and vertical lines highlighting their positions within the genes below. A regional *locuszoom* plot **E** displaying the LD pattern between the top variant and the other variants within the region

*The manhattan* function displays a Manhattan plot with the chromosomal position of genetic variants on the x-axis versus the − log_10_(*P*) of the association statistic on the y-axis. It consists of a single panel in which white-coloured semi-transparent shades/rectangles are used to distinguish between the chromosomes (Fig. 1A). The transparency and colour of the shades/rectangles can be altered, which is useful for simultaneously displaying multiple GWAS results (Fig. 1B). The *manhattan* function is tailored to display whole-genome association results, although it can also be used to plot association results for a single chromosome or smaller genetic regions.

The *regionplot* is customised for displaying association results for smaller genetic regions and consists of three panels: overview, main, and gene plots (Fig. 1C, D). The overview displays the association results over the entire chromosome with a red rectangle indicating the region of the chromosome that is displayed on the main and gene plots. If the region on display is small, the red rectangle may appear as a line. The user can specify the region to be displayed on the main and gene plots using variant ID (if included in the input dataset), gene name, or the genetic region represented as a chromosome, together with the start and stop positions (either as a single string or as three separate arguments). The *locuszoom* plot includes the main and gene plots and displays the regional association results coloured by the variant correlation (r^2^), which must be pre-calculated (for one of the variants against the rest) (Fig. 1E).

The graphical parameters used by the R packages *ggplot2* and *ggrepel* (e.g., *size, shape, colour, alpha, angle, nudge_x,* and *nudge_y*) can be passed to all *topr* plot functions to control the plot and variant label aesthetics.

### Main features

A useful feature is the retrieval and display of gene information from Ensembl (GRCh38 by default). Association peaks can be labelled with their nearest gene (or by the ID of the top variant) on any of the *topr* plots with the *annotate* argument (Fig. 1). The top variants within 1 Mb windows of the genome with association *p*-values below the given threshold (commonly referred to as index or lead variants) can be labelled with their nearest gene on genome and regional plots (*regionplot* and *locuszoom*), where the size of the 1 Mb window can be altered for sparser or denser labelling. Genes of interest are displayed at the bottom of the Manhattan plot to obtain a visual representation of their position relative to association peaks (Fig. 1A, B). A horizontal line, representing a significance threshold of − log10(5 × 10^−8^) is displayed on the Manhattan and regional plots by default. The line can be removed, altered, or multiple lines representing many different significance thresholds can be shown (Fig. 1A, C, E).

Other useful *topr* plot arguments are *vline* and *rsids*. With the former, the user can draw single or multiple vertical lines through the plots at given genetic positions, to obtain a better view of where individual variants are in relation to genes and other variants. The *rsids* argument is used to label one or more variants of interest by the variant identifier (ID). The *annotate_with_vline* and *rsids_with_vline* arguments can be used to synchronously label variants and highlight their positions using vertical lines.

A key *topr* feature is the simultaneous display of multiple association results on the same plot (Fig. 1B, D). Simultaneous display is useful for comparing association results of the same trait derived from different studies, or for viewing meta-analysis results along with the GWAS summary statistics used to generate them to obtain a visual overview of the contribution of each study to the combined *p*-value (Fig. 1B). Conventional plots show association p-values on the − log10 scale, increasing upward from the origin on the vertical axis. When displaying multiple association results, it can be helpful to duplicate the y-axis, with one set of values increasing upwards and another increasing downwards (the axis is reversed). With *topr*, the user can choose a plot with either a single y-axis or two y-axes and can specify how many GWAS summary statistics to show on the ‘top’ and on the ‘bottom’ with the *ntop* argument. When multiple datasets are displayed on the same plot, different *p*-value thresholds for annotations can be applied to each by assigning the annotate argument with a vector of *p*-values, where the first *p*-value in the vector applies to the first input dataset, and so on. Similarly, multiple datasets can be plotted using different point shapes, sizes, colours, and transparency (alpha) by providing a vector of values to the input arguments. Finally, *topr* also includes a few simple yet useful helper functions, such as *get_lead_snps, annotate_with_nearest_gene*, *get_gene_coords, get_snps_within_region, get_snpset,* and *get_overlapping_snps_by_pos,* to aid in the process of exploring, comparing, and analysing association results.

## Results and discussion

The functionality of *topr* is best described with examples of typical usage using the three inbuilt datasets (CD_UKBB, CD_FINNGEN, and UC_UKBB), where any association results with three columns representing variant positions (e.g., CHROM, POS) and *p*-values (*p*) could be used instead. The code used to create the figures and tables is available at the *topr* website.

### Visual overview of annotated GWAS results

When an analyst acquires new GWAS results, he or she typically wants an instant visual overview of the results to evaluate the association pattern and identify potential association peaks and nearby genes. Here, I use *topr’s* inbuilt dataset CD_UKBB as an example of the newly obtained GWAS results. To obtain an immediate visual overview of the results, *topr´s manhattan* function is called using the association results (CD_UKBB) as the sole input. By also passing the *annotate* argument with a *p*-value (e.g., annotate = 1e−09) to the *manhattan* function, the top variants within each 1 Mb window of the genome with *p*-values below the assigned *p*-value are labelled with their nearest gene from genome build GRCh38. If the input dataset already includes gene information (in a column called *gene, Gene_Symbol*, or *Gene_name*), *topr* will use that for labelling instead. The size of the 1 Mb window can be altered with the *region_size* argument for sparser or denser labelling of association peaks. Alternatively, the *annotate_with_vline* argument can be used to further highlight the annotated variants by drawing a dashed vertical line through their genomic positions on the plot. To extract the lead variants labelled on the plot, the *get_lead_snps* helper function is called with the CD_UKBB dataset as the input. The extracted variants can subsequently be annotated with their nearest gene using the *annotate_with_nearest_gene* function*,* which takes any R data frame, including one or more genetic positions (represented as CHROM and POS), as input and returns the overlapping or nearest gene. The number of variants labelled on Manhattan and regional plots is controlled with the *region_size* and *annotate* arguments, whereas the number of variants extracted with helper functions is controlled with the *region_size* and *thresh* arguments. In addition to labelling variants with their nearest gene on the plots, the genomic location of specific genes of interest can easily be highlighted at the bottom of the Manhattan plots by providing their names (in a vector in R) to the *highlight_genes* argument (e.g., *highlight_genes* = c(“IL23R”, “NOD2”)).

After visually examining the genome-wide association results, it is of interest to further explore regions around the association peaks (or other regions of interest). The *regionplot* function displays association results for smaller genetic regions defined by the gene name, variant identifier (ID), or the coordinates of the genetic region (provided by the user). For example, calling the *regionplot* function with the association results and the *gene* argument set to IL23R displays the results within a genetic region spanning the entire length of the gene, with an additional 100 kb extending in each direction. The *gene_padding* argument is used to alter the size of the extended region. The coordinates of the displayed region are always shown in the *regionplot* and can thus be easily copied and passed to the *get_snps_within_region* function to extract the displayed variants. Similar to the Manhattan plot, the top variants are labelled using the *annotate* and *annotate_with_vline* arguments, and the labelling density is controlled with the *region_size* argument. By default, the top variants are labelled with their variant identifier (ID) on regional plots and their nearest gene on Manhattan plots; however, this can be altered by assigning the *annotate_with* argument to either *the gene* or *ID*. Regional plots display exon structure on the gene plots for regions smaller than 1 Mb; otherwise, the gene structure is shown; however, this can be controlled with the *show_genes* argument.

When exploring regional association results, it is usually of interest to view the linkage disequilibrium (LD) pattern between the top variant and other variants in the region. This can be achieved using *topr’s locuszoom* function if the input dataset includes pre-calculated correlation scores (R2). The GTLD function in the GORpipe query language [12] was used to calculate the pairwise correlation scores between the top variant and other variants within the region previously shown on the *regionplot.* To display the regional correlation pattern between the variants, the regional dataset, including the pairwise correlation scores, is provided as the first and only argument for the *locuszoom* function. Additional input arguments for controlling the labelling density and plot aesthetics are optional and the same as for other *topr* plot functions.

### Comparison of test statistics in two GWAS results

**Fig. 2:**
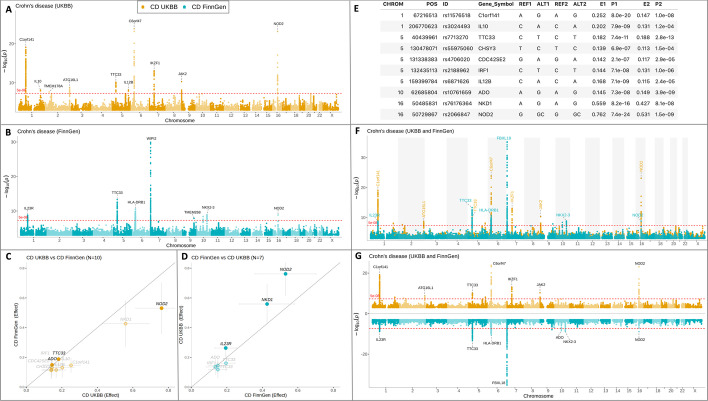
Comparison of GWAS results on Crohn´s disease from UK biobank (CD UKBB) and FinnGen (CD FinnGen). Association results on Crohn’s disease from UK biobank **A** are displayed on a Manhattan plot in orange with lead variants (*p* < 5 × 10^−8^) annotated with their nearest gene. Crohn’s disease association results from FinnGen **B** are shown in turquoise with lead variants annotated with their nearest genes. An *effectplot* **C** showing the effect sizes of the top variants in the CD UKBB dataset against the corresponding effect sizes in the CD FinnGen dataset. The effect sizes of the variants are represented by filled circles and labelled in black if the corresponding *p*-value for the variant in the second dataset (CD UKBB) is below (*p* < 5 × 10^−8^), otherwise an un-filled circle is displayed, and the gene label is shown in grey. The effectplot **D** showing the effect sizes of the top variants in the CD FinnGen dataset compared to the effect sizes of the same variants in the CD UKBB dataset. A *snpset* **E** created in *topr* using the g*et_snpset* function, to extract lead variants from the CD UKBB dataset and matching them with overlapping variants in the CD FinnGen dataset. Simultaneous and overlapping display **F** of the two Crohn’s disease datasets are shown in (**A**) and (**B**). Simultaneous and nonoverlapping display (**G**) of the two datasets

To further evaluate the newly obtained association results, it is useful to compare them with already available results on the same trait/phenotype from other studies or cohorts (if they exist). Here, I use the association results for Crohn’s disease from FinnGen, which also comes with the *topr* package (CD_FINNGEN), to compare with Crohn´s disease results from UKBB (CD_UKBB) used in the previous section. First, the two association results are displayed separately using the *manhattan* function with two different colours to distinguish the datasets and annotate and extract the lead variants in each dataset (Fig. 2A, B). Because the *manhattan* plot function only provides a comparative overview of association *p*-values, *topr* includes another function called *effectplot* to visually compare effect sizes and *p*-values of the lead/top variants overlapping in the two datasets (Fig. 2C, D). It requires two datasets (represented as a list in R), each containing the four columns (P, ALT, REF, OR/BETA) as input, or alternatively one dataset (a *snpset*) containing eight columns (P1, E1, ALT1, REF1, P2, E2, ALT2, REF2). If two datasets are provided as inputs, they are converted into a *snpset* in the *effectplot* function prior to plotting (Fig. 2E).

If the input datasets include odds ratios (OR), they are converted into beta estimates prior to generating the *snpset*. A *snpset* can also be generated directly from two datasets using the *get_snpset* helper function. A *snpset* is generated by 1) extracting the top/lead variants from the first input dataset (D1) and 2) retrieving overlapping variants (by position) from the second dataset (D2). Like in other *topr* helper functions, the number of variants extracted from the first datasets can be controlled with the *thresh* and *region_size* arguments. In the third step 3); overlapping variants are matched by their reference and alternative alleles (REF and ALT), and the variant effect is flipped in the second dataset (D2) if the reference allele matches the alternative allele in the first dataset (D1). If the alleles cannot be matched, they are not included in the *snpset.* The final step 4) in creating a *snpset* is to convert the effect sizes so that the effect is shown for the positive allele in the first input dataset (D1).

Each of the 4 steps above can be applied independently within *topr* using the helper functions; *get_lead_snps, match_by_pos, match_by_alleles* and *flip_to_positive_allele_for_dat1 (*see the *get_snpset_code* function for more details*)*. To get information on which variants are excluded and why in the process of creating a *snpset*, the *get_snpset* function is called with the *verbose* argument set to TRUE. The function returns three datasets, the *snpset* (matching variants), the variants from D1 with no overlapping variants in D2 (variants not found) and the variants in D1 that could be matched by position in D2 but not by allele (no allele match).

### Multiple GWAS results on the same plot

Instead of using multiple Manhattan or regional plots to compare association results by genome or region, they can be viewed simultaneously on a single Manhattan or regional plot using *topr.* Displaying the two Crohn’s disease datasets (CD_UKBB and CD_FINNGEN) on the same plot provides a clearer view of the overlapping peaks (Fig. 2F). By default, the two datasets are plotted using a single y-axis and are thus overlapping; however, this can be controlled with the *ntop* argument. For example, by setting the *ntop* argument to 1, the y-axis is duplicated with one result appearing at the top and the other at the bottom with a reversed y-axis (Fig. 2G). Displaying multiple association results on the same plot is especially useful when viewing meta-analysis results along with the association results used to generate them, to get a visual overview of dataset compatibility and contribution to respective association peaks. The association results shown in dark blue in Fig. 1A were generated by combining two Crohn’s disease datasets (CD_UKBB and CD_FINNGEN) in an inverse variance-based meta-analysis (4) implemented in the GORpipe query language [12]. It is evident from the plot, that most of the signals in the UK biobank dataset (orange) strengthen when combined with the summary statistics from FinnGen (turquoise) in the meta-analysis (darkblue). There are however also peaks where the combined *p*-value is larger than the single study *p*-value, indicating that these signals do not replicate in the other dataset. The compatibility of the two datasets can be further explored using *regionplot*, by zooming into regions centred on specific genes or variants. By calling *regionplot* with the *gene* argument set to IL23R and *annotate* to 1e−06, one gets a more detailed view of the region and the top variant in each dataset. The *rsids* or *rsid_with_vline* arguments can be used to label variants by their identifier (ID) in all the dataset on display, and their position on the plot can be controlled with the *nudge_x* argument, which can be assigned with either a single value or a vector of multiple values.

### Control over plot aesthetics

**Fig. 3:**
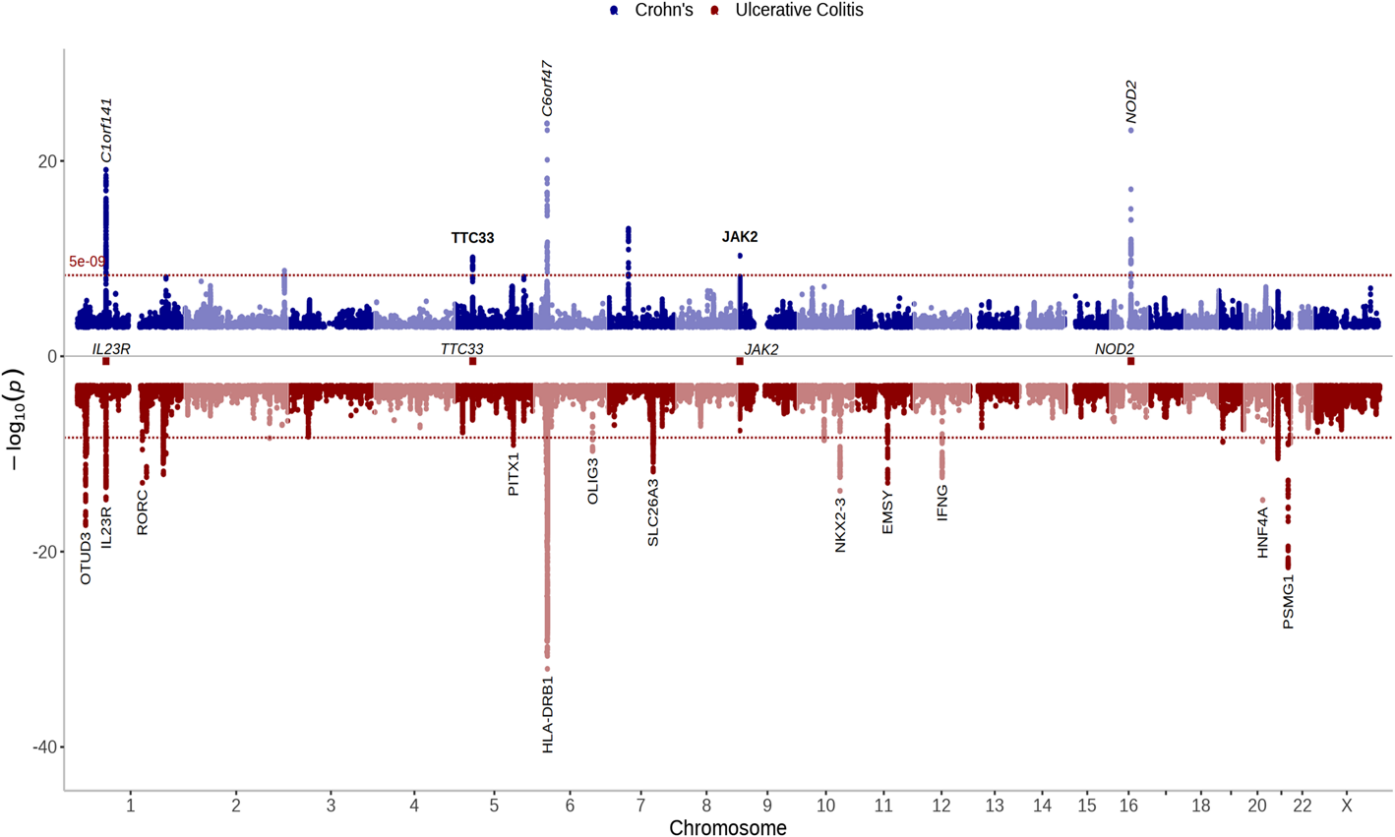
Manhattan plot generated with *topr* using different shades of the same colour to distinguish between chromosomes. Association results from UK biobank on Crohn’s are displayed in dark blue at the top and association results for Ulcerative colitis are shown in dark red at the bottom. Association peaks are annotated with their nearest genes using different *p*-value thresholds, angles (0 and 90) and varying font-faces (bold, plain, and italic)

The most common *ggplot* and *ggrepel* arguments can be used with *topr*. The user has full control over the plot point and label aesthetics (e.g., colour, size, shape, transparency, font-face, angle, and position), as well as labelling thresholds and density, where different values can be assigned (with a vector of values) to multiple datasets displayed on the same plot (Fig. 3). For example, on the same plot, the user can label the top variant within each 100 kb window with *p*-values below 1 × 10^12^ in one dataset and the top variant within each 10 Mb window with *p*-values below 1 × 10^8^ in another. Different plot points, shapes, sizes, and transparencies can be assigned to the two datasets and their top variants are labelled using distinct angles or positions in relation to the variants (using *nudge_x* and *angle* arguments). A legend figure is added by default to all Manhattan and regional plots displaying more than one dataset. The position of the legend can be altered, or it can be omitted from the plots. Furthermore, the user can change the colour and/or transparency of the shades/rectangles used to distinguish between the chromosomes in the Manhattan plot.

## Conclusion

*topr* is an R package tailored for viewing, analysing, and comparing phenotype/genotype association results. Distinctive *topr* features are the gene annotation functionality and simultaneous display of multiple GWAS results on single or duplicate y-axis along with the ability to further zoom in on the data by gene name, variant identifier (ID) or genetic region. The user can visualise, annotate, and simultaneously compare multiple datasets before creating publication ready plots that highlight genes and/or variants of interest. In addition to plot functions, *topr* includes helper functions to aid in data analysis, such as extracting the top/lead variants, annotating variants with their nearest gene, and finding overlapping variants in other datasets. The *topr* package was created to make it easy to quickly view, annotate, and compare elegant plots of association results, at the same time as providing a flexible tool with multiple features to suit the need of different users. Future development of *topr* will focus on inclusion and display of regulatory elements (e.g. enhancers, promoters and transcription factor binding sites) and expression quantile trait loci (eQTLs) to aid in the mapping of non-coding GWAS signals to affected genes. Furthermore, to facilitate the increased popularity of gene based association approaches, future versions will include the display of results from variant aggregation methods, such as transcriptome-wide associatoion studies (TWAS) and sequence kernel association test (SKAT).

## Data Availability

*topr* is freely available on the Comprehensive R Archive Network (http://cran.r-project.org/package=topr). The source code and data is available at GitHub (https://github.com/totajuliusd/topr). Project name: topr, Project home page: http://cran.r-project.org/package=topr and https://github.com/totajuliusd/topr, Project documentation: https://github.com/totajuliusd/topr, Operating systems: Linux, Mac OS, Windows, Programming language: R, Other requirements: R-3.5.0 or higher. Licence: LGPL (> 3), Any restrictions to use by non-academics: none.

## Abbreviations

GWAS: Genome wide association studies
SNP: Single nucleotide polymorphism
Mb: Million base pairs
CD: Crohn’s disease
UC: Ulcerative colitis
IBD: Inflammatory bowel disease
